# Exosome-Like Vesicles from *Lithospermum erythrorhizon* Callus Enhanced Wound Healing by Reducing LPS-Induced Inflammation

**DOI:** 10.4014/jmb.2410.10022

**Published:** 2024-11-28

**Authors:** Hyeonoh Kim, Hyun-young Shin, Mira Park, Keunsun Ahn, Seung-Jin Kim, Sang-Hyun An

**Affiliations:** 1 Preclinical Research Center, Daegu Gyeongbuk Medical Innovation Foundation (K-MEDI hub), Daegu 41061, Republic of Korea; 2 Research Institute, Sphebio Co., Ltd., Seoul 04796, Republic of Korea

**Keywords:** *Lithospermum erythrorhizon* exosome-like vesicles, Lipopolysaccharide (LPS)-induced wounds, wound healing, anti-inflammation effects

## Abstract

*Lithospermum erythrorhizon* (LE), a medicinal plant from the Boraginaceae family, is traditionally used in East Asia for its therapeutic effects on skin conditions, including infections, inflammation, and wounds. Recently, the role of extracellular vesicles (EVs) as mediators of intercellular communication that regulate inflammation and promote tissue regeneration has garnered increasing attention in the field of regenerative medicine. This study investigates exosome-like vesicles derived from LE callus (LELVs) and their potential in enhancing wound healing. *In vitro* studies using normal human dermal fibroblasts (NHDFs) demonstrated that LELVs significantly improved cell viability, proliferation, and wound closure, while also enhancing collagen type I synthesis, indicating anti-inflammatory and regenerative properties. For in vivo analysis, LELVs were applied to lipopolysaccharide (LPS)-induced wounds in mice, where wound healing progression was monitored over 14 days. LELV-treated wounds exhibited accelerated re-epithelialization, reduced inflammation, and improved tissue remodeling, with histological analysis revealing enhanced collagen deposition and reduced inflammatory cell infiltration. These results highlight the ability of LELVs to modulate the inflammatory response and promote wound healing. With their natural origin, low immunogenicity, and ease of production, LELVs represent a promising alternative to synthetic treatments for inflammation-associated skin injuries and hold significant potential for clinical applications in wound care.

## Introduction

The skin, the largest organ in mammals, plays an indispensable role in maintaining homeostasis, offering protection against environmental assaults such as pathogens, ultraviolet (UV) radiation, and physical trauma. Its primary function as a barrier is crucial for preventing excessive water loss and microbial invasion, thus preserving the body’s internal environment. When the skin is injured due to trauma, burns, or surgery, it initiates a complex and dynamic wound healing process that progresses through four overlapping stages: hemostasis, inflammation, proliferation, and tissue remodeling [1-3]. The efficient orchestration of these stages is vital to restoring the skin's structural and functional integrity.

Inflammation, the second phase of wound healing, is pivotal in clearing pathogens and cellular debris from the wound site. This inflammatory phase is marked by the recruitment of immune cells such as neutrophils, macrophages, and lymphocytes, which help to initiate the subsequent tissue repair processes [4, 5] However, when inflammation is excessive or dysregulated, it can lead to delayed wound healing, tissue damage, or chronic wounds, particularly in conditions associated with bacterial infections, immune dysfunctions, or metabolic diseases like diabetes [6]. Lipopolysaccharide (LPS), a component of Gram-negative bacterial membranes, is often used in experimental models to simulate inflammation-related delays in wound healing by inducing a robust immune response [7-9].

Recent advances in regenerative medicine have highlighted the significance of extracellular vesicles (EVs) as mediators of intercellular communication, particularly in modulating inflammation and promoting tissue regeneration [10, 11]. EVs are lipid bilayer-enclosed particles that carry proteins, lipids, and nucleic acids, and capable of transferring bioactive molecules between cells, influencing various biological processes including immune modulation and tissue repair [12]. Among EVs, exosome-like vesicles have garnered attention for their ability to regulate inflammation and promote wound healing through the delivery of growth factors, miRNAs, and anti-inflammatory proteins to target cells [13]. While EVs derived from mammalian cells have been extensively studied, plant-derived EVs have emerged as promising candidates for therapeutic applications due to their low immunogenicity, ease of production, and potential scalability [14, 15].

*Lithospermum erythrorhizon* (LE), a medicinal plant from the Boraginaceae family, has been traditionally used in East Asian medicine for treating skin ailments such as wounds, infections, and inflammatory skin conditions. The plant’s bioactive compound, shikonin, and its derivatives are known for their broad pharmacological properties, including anti-inflammatory, antimicrobial, and wound-healing effects [16, 17]. Shikonin has been shown to inhibit angiogenesis, reduce oxidative stress, and regulate immune responses, making it a valuable therapeutic agent for various skin disorders [18, 19]. Despite its traditional use and the well-documented pharmacological activities of LE extracts, the potential of exosome-like vesicles derived from *Lithospermum erythrorhizon* callus (LELVs) in wound healing and inflammation modulation has not been fully explored.

This study aims to isolate and characterize LELVs and evaluate their therapeutic potential in enhancing wound healing, particularly under inflammatory conditions. We hypothesize that LELVs, enriched with bioactive components from *L. erythrorhizon*, can attenuate LPS-induced inflammation, promote dermal fibroblast proliferation, and facilitate faster wound closure. To test this hypothesis, we employed both in vitro studies using normal human dermal fibroblasts (NHDFs) and *in vivo* models of LPS-induced skin wounds in mice. The findings from this study could offer new insights into the development of natural, plant-derived extracellular vesicles as therapeutic agents for inflammation-associated wounds and skin regeneration.

## Materials and Methods

### Isolation of LELV

A two-week suspension culture of *Lithospermum erythrorhizon* (LE) callus was conducted. The callus was separated from the medium and ground in 1X phosphate-buffered saline (PBS). The homogenate was mixed with the harvested medium, filtered through a 30 μm Miracloth filter (Millipore, USA), and then centrifuged at 3,500 ×*g* for 30 min. The supernatant was collected to remove cell residue, and this step was repeated to ensure purity. To isolate LELVs, the supernatant was passed through a 0.45 μm membrane filter (Hyundai Micro, Republic of Korea). Tangential flow filtration (TFF) was then performed using a 100 kDa membrane. The exosomes were sterilized with a 0.2 μm bottle-top filter (Corning, USA) and stored at -80°C until use.

### Characterization of LELV

The isolated LELVs were characterized using nanoparticle tracking analysis (NTA) equipment (*ZetaView*, Particle Metrix, Germany) to determine their size and concentration. Calibration was performed using 100 nm polystyrene beads (Applied Microspheres, Germany). To achieve the optimal particle concentration for analysis, LELVs were diluted 1:1000 in filtered PBS. The experiment was configured to measure 200 particles per screen. The diluted sample was loaded into the chamber, and eleven measurements were taken at different positions in the cell at 25°C to ensure reproducibility. The measurements followed the scatter analysis standard operating procedure (SOP), and the data were processed using *ZetaView* version 8.04.04 SP2 software. For transmission electron microscopy (TEM) analysis, 5 μl of LELVs were pipetted onto a carbon-coated grid and incubated for 1 min. The sample was stained with 2% uranyl acetate for 20 sec and observed under a transmission electron microscope at an accelerating voltage of 80 kV (JEM-1010, Jeol, Japan).

### Cell Culture

Normal human dermal fibroblasts (NHDF PromoCell, Germany) and mouse macrophages (Raw264.7, Korean Cell Line Bank, Republic of Korea) were cultured in Dulbecco’s Modified Eagle’s medium (Hyclone, USA) supplemented with 10% fetal bovine serum (FBS) (Hyclone, USA) or exosome-depleted FBS (Gibco, USA) and 1% penicillin-streptomycin (Welgene, Republic of Korea). The cells were maintained at 37°C in a humidified atmosphere containing 5% CO_2_.

### Cell Viability and Proliferation Assay

The effects of LELVs on NHDFs viability and proliferation were assessed using the Cell Counting Kit-8 (CCK-8) assay (Dojindo Molecular Technologies, Inc., Japan), according to the manufacturer's instructions. NHDFs were seeded at a density of 4 × 10^3^ cells per well in a 96-well plate and incubated for 24 h at 37°C in a humidified atmosphere containing 5% CO_2_. The following day, NHDFs were treated with LELVs at concentrations of 1 × 10^8^, 1×10^9^, and 1 × 10^10^ particles/ml in DMEM supplemented with exosome-depleted FBS. The cell viability was assessed at 24 and 48 h post-treatment by adding the CCK-8 solution directly to the wells and incubating for an additional period, as per the assay protocol. The absorbance was recorded at a wavelength of 450 nm using a microplate reader to determine cell viability.

### In Vitro Scratch Wound Healing Assay

For wound healing assessment, NHDFs were seeded at 2 × 10^4^ cells per well in a scar block (Ibidi, Germany) and incubated for 24 h at 37°C with 5% CO_2_. The cells were washed with PBS and treated with LELVs (1 × 10^8^, 1 × 10^9^, and 1×10^10^ particles/ml) or a buffer control in DMEM with exosome-depleted FBS. Wound closure was photographed under a light microscope at 0 and 24 h post-scratch and analyzed using the Image J software. The percentage of wound closure was calculated and normalized to that of the control group.

### Anti-Inflammatory Assay

The anti-inflammatory effect of LELVs was assessed by measuring TNF-α production. Mouse macrophages (Raw264.7) were seeded at 1 × 10^5^ cells per well in a 24-well plate and incubated for 24 h at 37°C with 5% CO_2_. Following incubation, the cells were stimulated with 1 μg of LPS for 4 h to induce inflammation. Subsequently, the cells were treated with LELVs at concentrations of 1 × 10^8^, 1 × 10^9^, and 1×10^10^ particles/ml for an additional 20 h. The levels of TNF-α in the supernatant were quantified using an enzyme-linked immunosorbent assay (ELISA) kit (R&D Systems, USA).

### Collagen Synthesis Assay

To evaluate the impact of LELVs on collagen synthesis, NHDFs were seeded at 3×10^4^ cells per well and pre-cultured. After 48 h of treatment with various LELV concentrations, procollagen type I synthesis was assessed using an ELISA kit specific for human collagen type I (R&D Systems, USA). The amount of collagen synthesized was normalized to the total protein content.

### Cellular Uptake of LELV

LELVs were assessed using the PKH67 Green Fluorescent Cell Linker Kit (Sigma Aldrich, USA) following the manufacturer’s protocol. Stained LELVs were mixed with Diluent C and the PKH67 dye solution and incubated on ice for 5 min, and the reaction was stopped with PBS containing 1% bovine serum albumin (BSA). The labeled LELVs were concentrated using a 10 kDa MWCO filter column (Amicon, USA), washed three times with PBS, and centrifuged at 3,000 ×*g* for 15 min. NHDFs were seeded at a density of 2.5 × 10^4^ cells per well on 18 × 18 mm round coverslips and incubated at 37°C in a 5% CO_2_ incubator for 24 h. The next day, the cultured fibroblasts were co-incubated with PKH67-labeled or unlabeled LELVs for 4 and 24 h. After co-incubation, the cells were washed with PBS, fixed with 4% paraformaldehyde for 30 min, and stained with 4,6-diamidino-2-phenylindole (DAPI).

### In Vivo Wound Healing Experiments

All animal procedures were approved by the Institutional Animal Care and Use Committee of the Daegu-Gyeongbuk Medical Innovation Foundation (K-MEDI Hub) (IACUC approval number: KMEDI-23081601-00). Seventy 5-week-old male ICR mice, with an average weight of 20–25 g, supplied by KOATECH (KOATECH Corporation, Korea), were randomly assigned into four groups: control, LPS-only, LELV-only, and LPS + LELV.

*Klebsiella pneumoniae*-derived LPS (Sigma-Aldrich, L4268) was administered at a dose of 0.5 mg/kg via subcutaneous (SC) injection to the LPS and LPS + LELV groups 24 h prior to wound formation. Mice were anesthetized, and the hair on their backs was shaved using clippers. A circular full-thickness skin wound (10 mm diameter) was created on the dorsal midline using a biopsy puncher. The LELV group and the LPS + LELV group were treated with 1 × 10^10^ particles/ml of LELVs. Digital photographs of the wounds were taken on days 0, 3, 5, 7, 10, and 14, with a standard ruler included for scale. The wound size was measured using ImageJ software (National Institutes of Health, USA), and the percentage reduction in wound area was calculated relative to the initial wound size.

### Histological Staining

All animals were sacrificed after 7 and 14 days of healing. The skin at the wound site of each group was excised and fixed for histological evaluation. The area most affected by each wound was selected for analysis. Tissue sections were fixed overnight in 10% buffered formalin at room temperature, cut horizontally, dehydrated, and transferred to 70% ethanol for 48 h before being embedded in paraffin. Sections (5 μm) were stained with hematoxylin and eosin (H&E) and Masson’s trichrome (MT) for histological analysis. Slide images were captured using a Pannoramic 250 Flash III scanner (3DHISTECH Kft, Hungary).

### Statistical Analysis

All experiments were conducted in triplicate, and data are presented as the mean ± standard deviation (SD). Comparisons between two groups were performed using the Student’s t-test, and comparisons among more than two groups were conducted using ANOVA. A *p* < 0.05, *p* < 0.01, or *p* < 0.001 was considered statistically significant.

## Results

### Isolation and Characterization of LELVs from *Lithospermum erythrorhizon* Callus

**Fig. 1:**
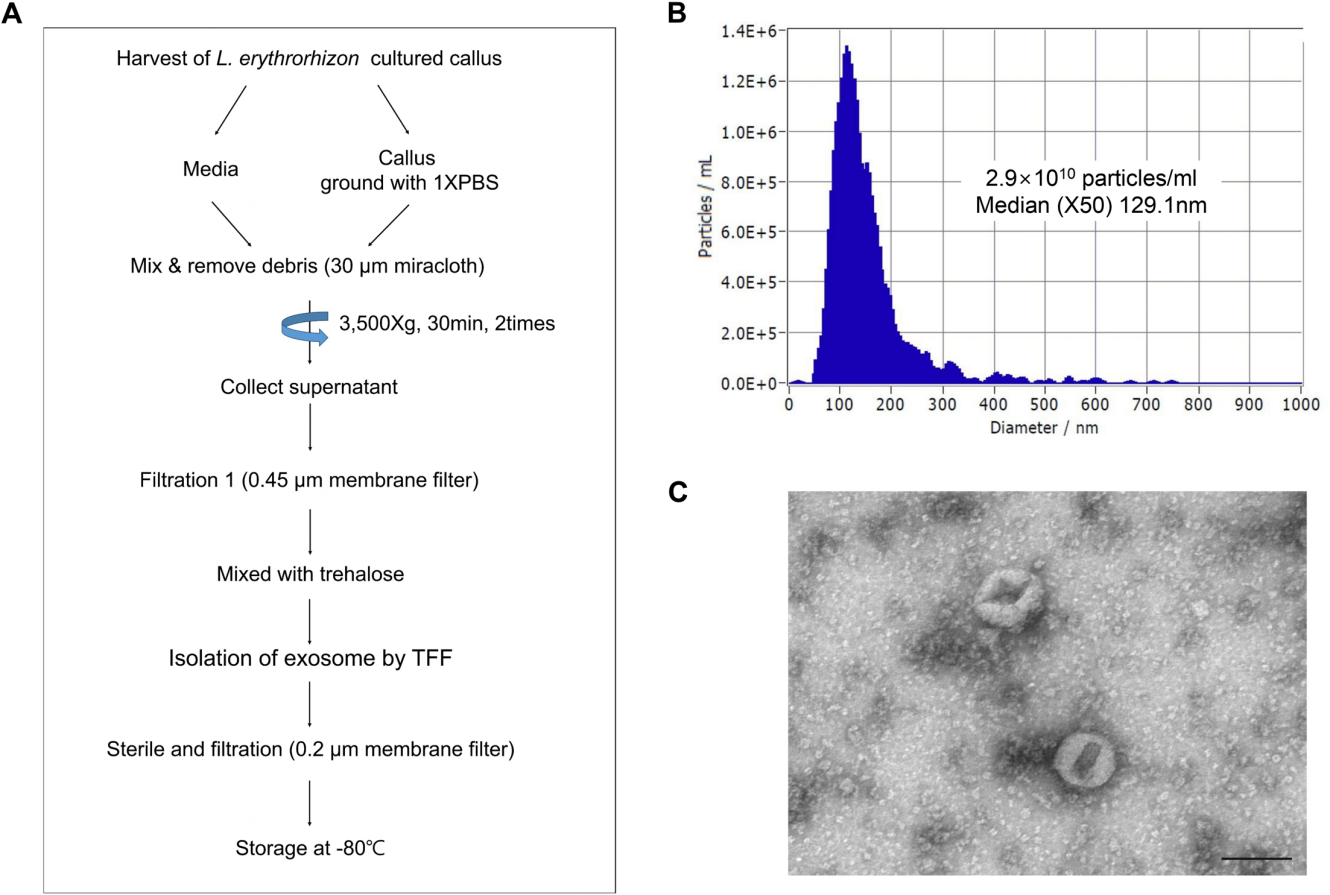
Isolation and characterization of exosome-like vesicles from *Lithospermum erythrorhizon* callus (LELVs). (**A**) Overview of the LELV isolation workflow, including suspension culture of the callus, separation from the medium, grinding in PBS, and filtration through a 30 μm Miracloth filter, followed by sequential centrifugation and tangential flow filtration (TFF) using a 100 kDa membrane. (**B**) Nanoparticle tracking analysis (NTA) conducted to determine the size distribution and concentration of LELVs, with the sample prepared by diluting in PBS and calibrated using 100 nm polystyrene beads. (**C**) Transmission electron microscopy (TEM) imaging was performed to observe the morphology of LELVs. Scale bar: 100 nm.

This study first aimed to isolate and characterize exosome-like vesicles (LELVs) from *Lithospermum erythrorhizon* callus (Fig. 1A). NTA analysis confirmed that the LELVs had a size distribution ranging from 89 to 200 nm, with an average diameter of 129 nm and a concentration of 2.3 × 10^10^ particles/ml (Fig. 1B). TEM images showed vesicles with a cup-shaped morphology typical of exosome-like particles (Fig. 1C). These findings demonstrate the successful isolation of vesicles with the expected characteristics, ensuring their suitability for use in biological assays.

### Cytotoxicity of LELVs in NHDFs

**Fig. 2:**
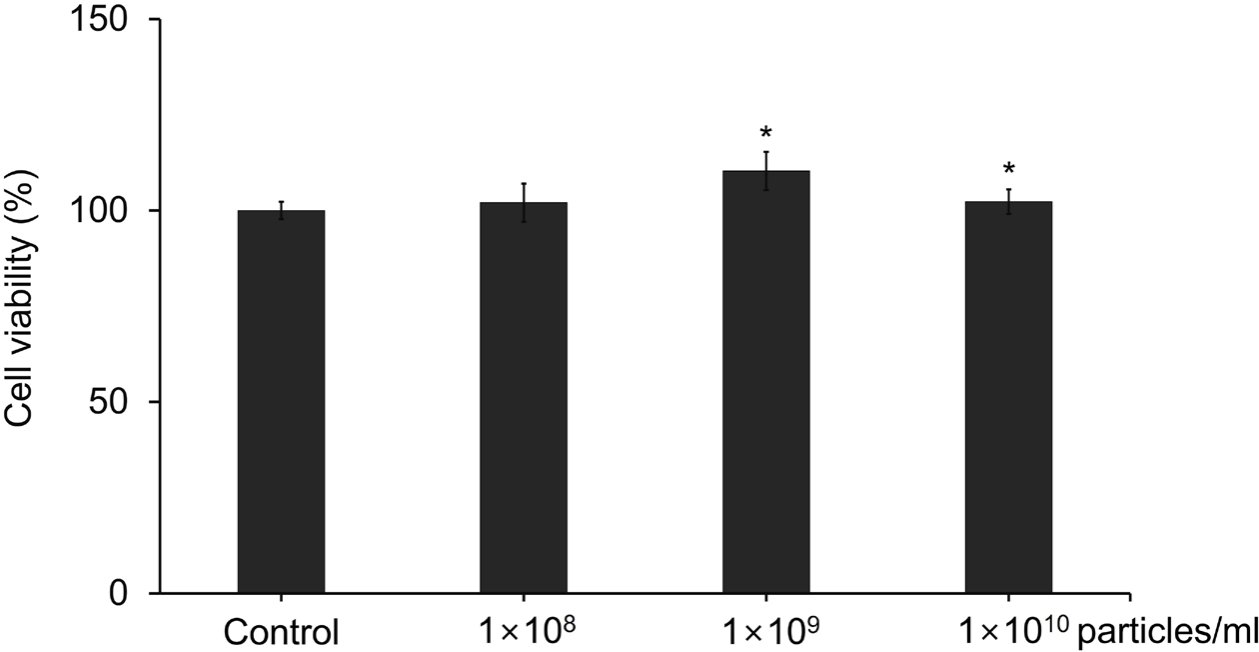
Cytotoxicity assessment of LELVs in NHDFs. Normal human dermal fibroblasts (NHDFs) were treated with LELVs at concentrations of 1 × 10^8^, 1 × 10^9^, and 1 × 10^10^ particles/ml for 24 h. Cell viability was evaluated using the CCK-8 assay, with absorbance measured to determine cytotoxic effects. Data are expressed as the mean ± S.D. *p < 0.05 compared with the control group.

NHDFs were treated with LELVs at concentrations of 1 × 10^8^, 1 × 10^9^, and 1 × 10^10^ particles/mL for 24 h. Cell viability was measured using the CCK-8 assay. All treated groups maintained cell viability above 95%, indicating no cytotoxic effects. Treatments with 1 × 10^9^ and 1 × 10^10^ particles/ml slightly increased cell viability compared to the control group (*p* < 0.05), suggesting that LELVs may positively influence cellular function (Fig. 2). These findings confirm that LELVs are non-toxic and suitable for further biological assays.

### LELVs Promote Fibroblast Proliferation

**Fig. 3:**
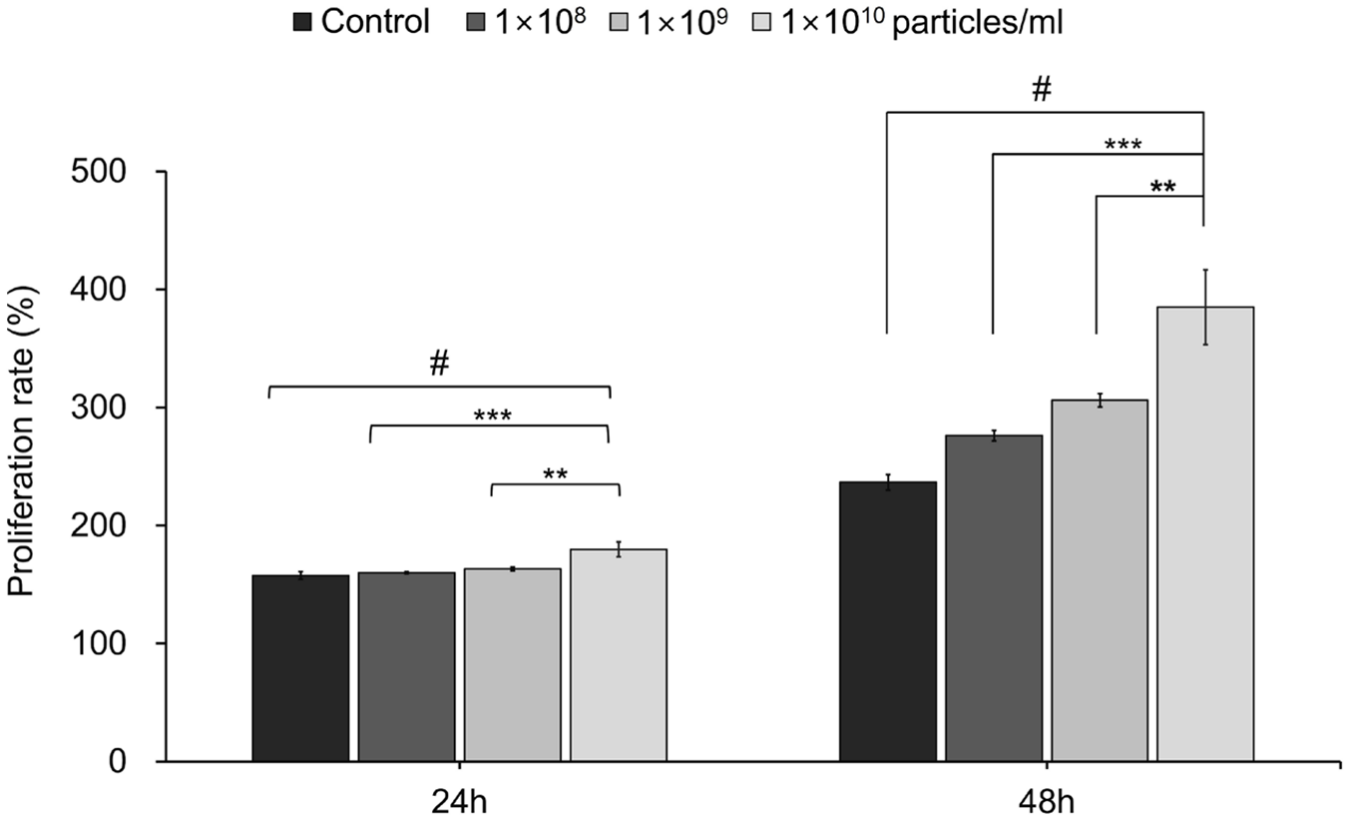
Proliferation of NHDFs following LELV treatment. NHDF proliferation was measured using the CCK-8 assay at 24 and 48 h post-treatment with LELVs at concentrations of 1 × 10^8^, 1 × 10^9^, and 1 × 10^10^ particles/ml. Data are expressed as the mean ± S.D. *p < 0.01, ***p < 0.001, # *p* < 0.0001 compared with the control group.

To determine the effect of LELVs on cell proliferation, NHDFs were treated with various concentrations of LELVs for 24 and 48 h. After 24 h, a significant increase in proliferation was observed only at the highest concentration (1 × 10^10^ particles/ml), resulting in a 13.7% increase compared to the control (*p* < 0.0001) (Fig. 3). At 48 h, a clear dose-dependent effect was evident: proliferation increased by 16.5%, 29.3%, and 62.7% for 1 × 10^8^, 1×10^9^, and 1 × 10^10^ particles/ml, respectively (*p* < 0.0001). These results indicate that longer exposure to LELVs enhances fibroblast proliferation, potentially accelerating the initial stages of wound healing through cell growth promotion.

### LELVs Enhance Wound Closure in NHDFs

**Fig. 4:**
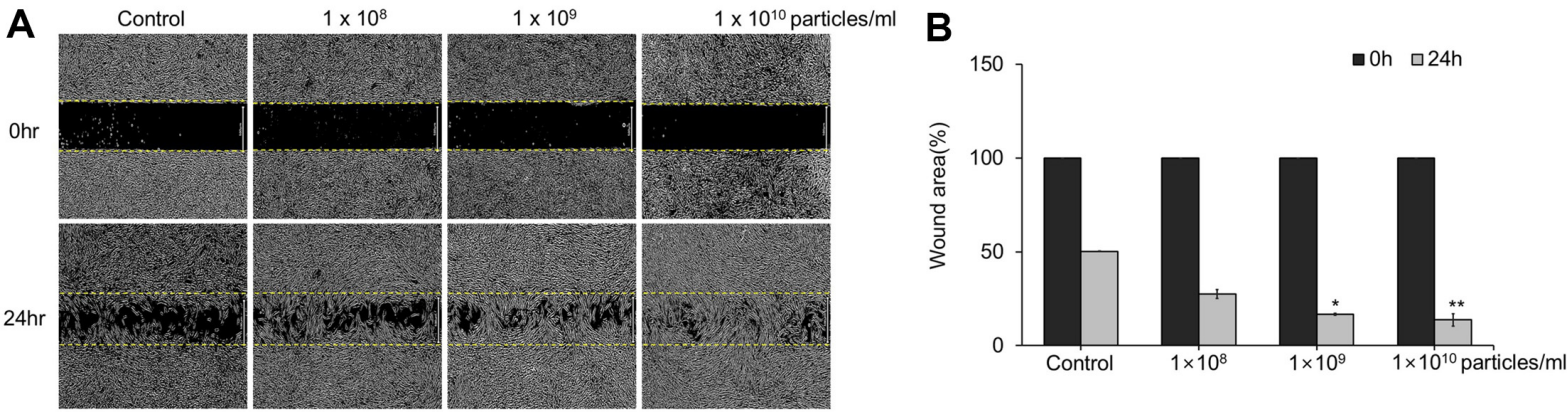
Wound closure assay in NHDFs treated with LELVs. (**A**) NHDF monolayers were scratched and treated with LELVs at 1 × 10^8^ , 1 × 10^9^ , and 1 × 10^10^ particles/ml, (**B**) with wound closure photographed and measured at 0 and 24 h. Data are expressed as the mean ± S.D. *p < 0.05, **p < 0.001 compared with the control group.

The wound-healing potential of LELVs was assessed using a scratch assay on NHDF monolayers. Artificial wounds were treated with varying concentrations of LELVs, and closure was monitored over 24 h. As shown in (Fig. 4), wound closure was significantly enhanced in a dose-dependent manner, with 47.7%, 66.9% (*p* < 0.05), and 76.5% (*p* < 0.05) closure observed for 1 × 10^8^, 1×10^9^, and 1 × 10^10^ particles/ml, respectively. Microscopic analysis confirmed that LELV-treated cells migrated more rapidly toward the wound area compared to untreated cells, suggesting that LELVs enhance fibroblast motility, a key factor in tissue repair.

### Anti-Inflammatory Effect of LELVs in LPS-Stimulated Macrophages

**Fig. 5:**
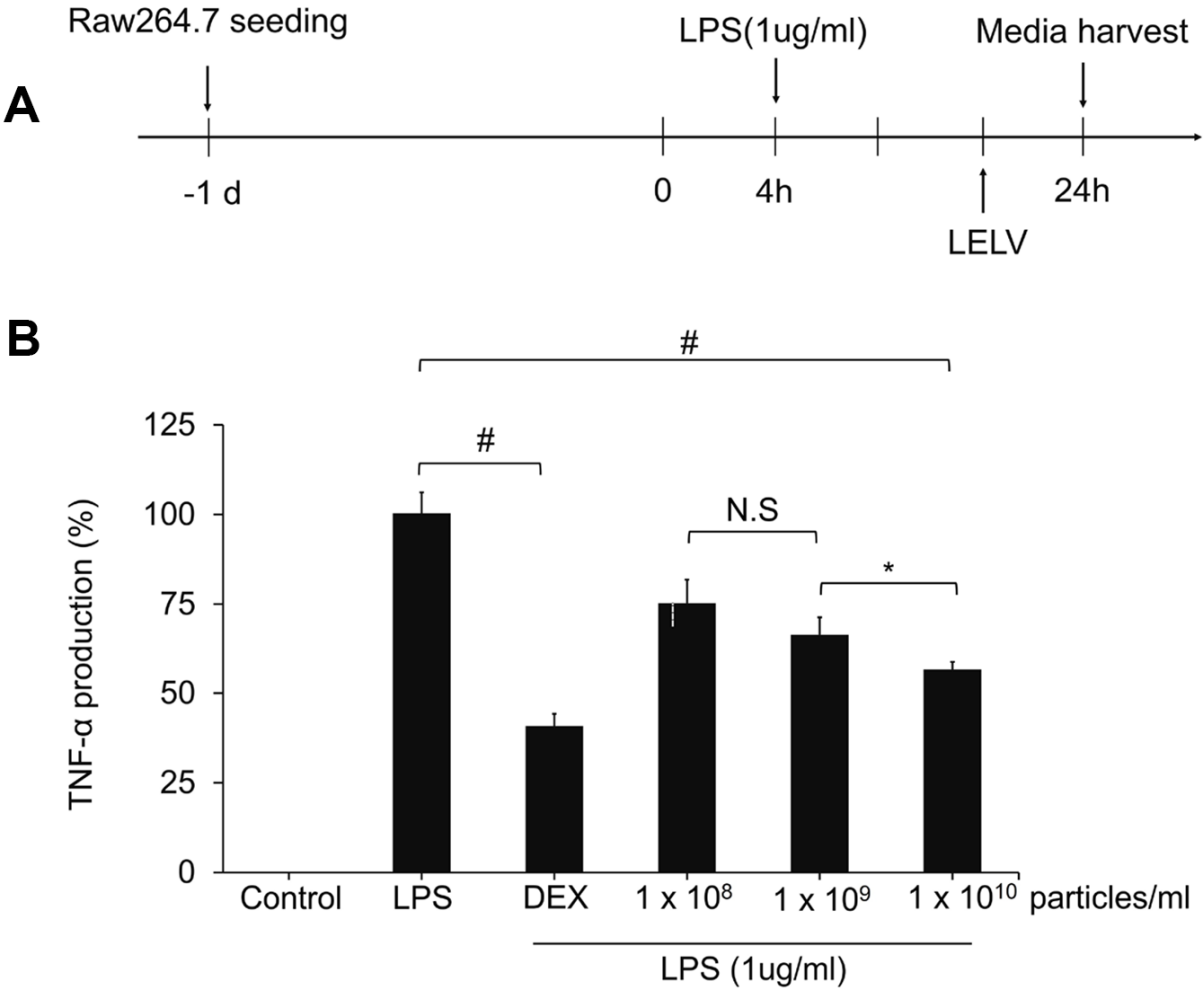
Anti-inflammatory effects of LELVs on LPS-stimulated RAW 264.7 macrophages. (**A**) Experimental setup showing macrophage stimulation with LPS (1 μg/ml), followed by treatment with LELVs or dexamethasone (DEX) as a control. (**B**) TNF-α levels were measured using ELISA following LELV treatment. Data are expressed as the mean ± S.D. *p < 0.05, # *p* < 0.0001, NS, non-significant compared with the LPS-only group.

To evaluate the anti-inflammatory effects of LELVs, we used an LPS-induced inflammation model. LPS stimulation resulted in a significant increase in TNF-α levels, indicating a robust inflammatory response. However, treatment with LELVs effectively reduced TNF-α production in a concentration-dependent manner. At the highest concentration (1 × 10^10^ particles/ml), TNF-α levels decreased by 43.5% compared to the LPS-only group (*p* < 0.0001) (Fig. 5). These results demonstrate that LELVs possess strong anti-inflammatory properties, suggesting their potential for treating inflammation-related wounds.

### LELVs Enhance Collagen Type I Synthesis in NHDFs

**Fig. 6:**
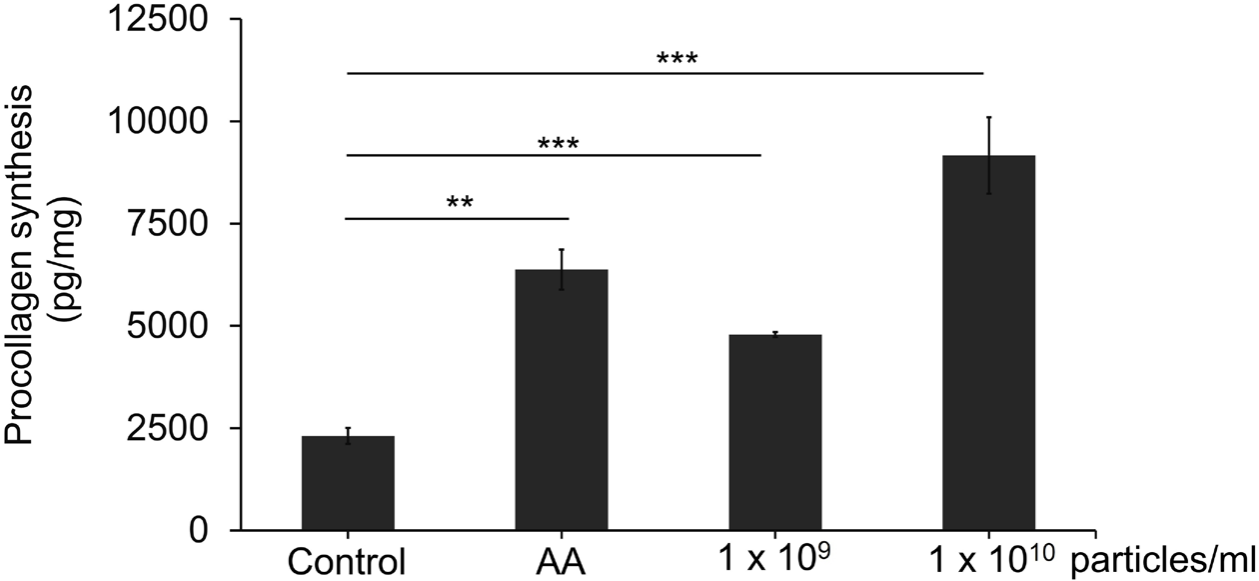
Collagen type I synthesis in NHDFs treated with LELVs. NHDFs were exposed to LELVs at 1 × 10^9^ and 1 × 10^10^ particles/ml for 48 h, with ascorbic acid (AA) as a positive control. Collagen levels were quantified using an ELISA kit specific to procollagen type I. Data are expressed as the mean ± S.D. **p < 0.01, ***p < 0.001 compared with the control group.

Since collagen production is essential for tissue regeneration, we investigated the effect of LELVs on collagen type I synthesis. NHDFs were treated with LELVs for 48 h, and collagen levels were measured using an ELISA kit. As shown in (Fig. 6), LELVs significantly increased collagen production in a dose-dependent manner, with a 106.8% increase at 1 × 10^9^ particles/ml (*p* < 0.0001) and a 296.0% increase at 1 × 10^10^ particles/ml (*p* < 0.001) compared to the control. These results suggest that LELVs enhance extracellular matrix formation, which is crucial for wound healing and tissue remodeling.

### Cellular Uptake of LELVs by NHDFs

**Fig. 7:**
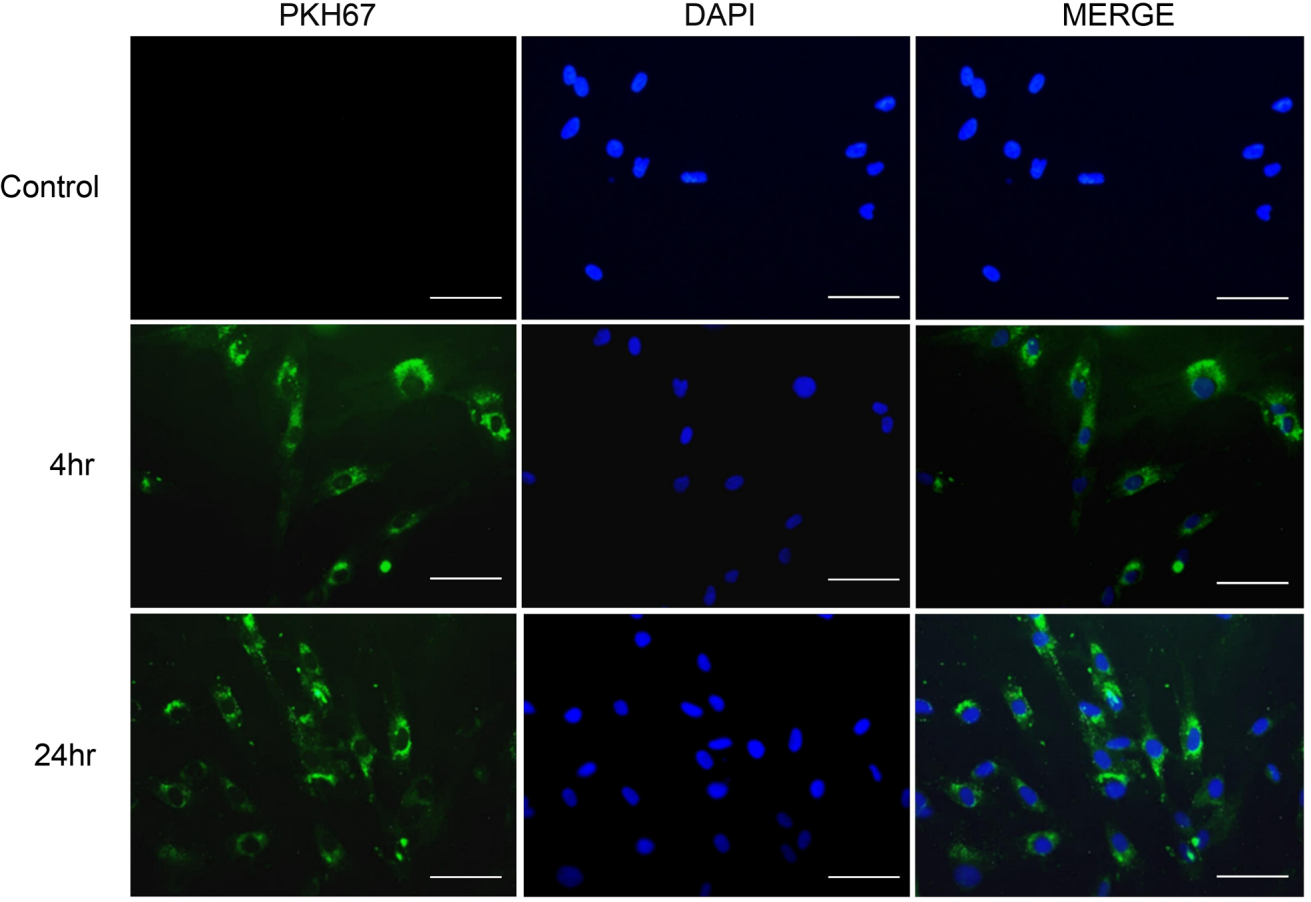
Cellular uptake of LELVs in NHDFs visualized by fluorescence microscopy. LELVs (1 × 10^8^ particles/ml) were labeled with PKH67 dye and incubated with NHDFs for 4 and 24 h. Fluorescence microscopy confirmed LELV internalization. Control cells treated with unlabeled LELVs showed no fluorescence. Scale bar: 10 μm.

To confirm that LELVs are internalized by fibroblasts, we labeled the vesicles with PKH67 dye and incubated them with NHDFs for 4 and 24 h. Fluorescence microscopy showed that PKH67-labeled LELVs were visible inside the cells, with more intense fluorescence observed at 24 h (Fig. 7). This successful uptake demonstrates that LELVs can efficiently deliver bioactive molecules to target cells, supporting their therapeutic potential.

### LELVs Accelerate Wound Healing in LPS-Induced Mice

**Fig. 8:**
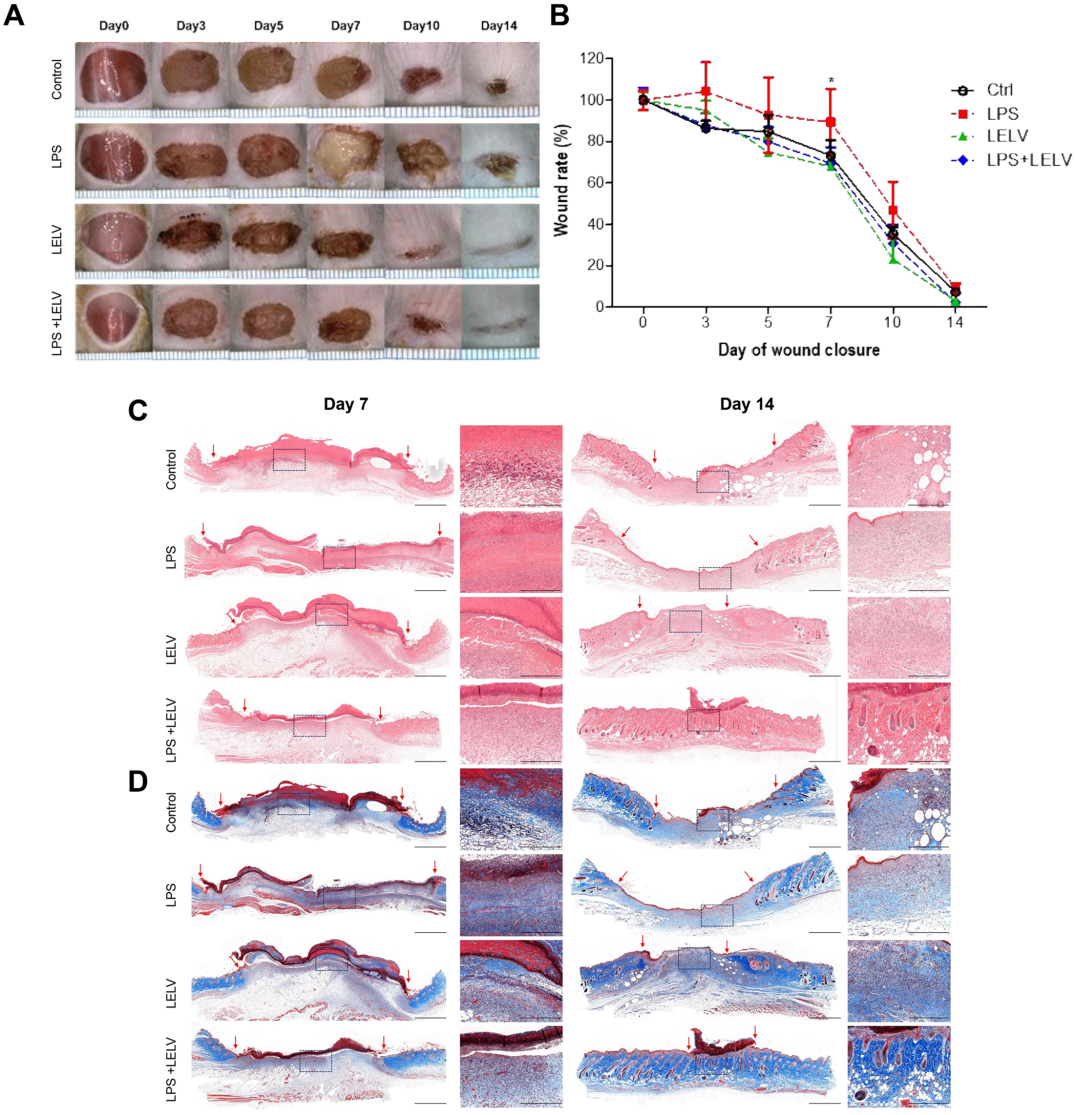
Wound healing in a mouse model with LPS-induced delayed healing treated with LELVs. (**A**) Wound images from mice receiving LPS (0.5 mg/kg, SC injection), LELVs (1 × 10^10^ particles/ml), or both over a 14 day period. (**B**) Wound size measurement on day 14. (**C**) H&E staining of wound tissue on dayss 7 and 14. (**D**) Masson’s trichrome staining for collagen assessment. Scale bars: low power field, 500 μm; high power field, 50 μm. Data are expressed as the mean ± S.D. *p < 0.05 compared with the control group.

The therapeutic potential of LELVs was further evaluated in an in vivo wound healing model using LPS to induce delayed healing. Mice were divided into four groups: control, LPS-only, LELV-only, and LPS + LELV groups. As shown in (Fig. 8A and 8B), LELV-treated groups exhibited significantly faster wound closure compared to the LPS-only group, with a 32% greater reduction in wound size by day 7 (*p* < 0.05).

Histological analysis with H&E and Masson’s trichrome staining confirmed that LELV-treated wounds had reduced inflammatory cell infiltration and enhanced collagen deposition (Fig. 8C and 8D). These findings indicate that LELVs accelerate wound healing by modulating inflammation and promoting tissue remodeling, even under inflammatory conditions.

## Discussion

In this study, we investigated the potential of isolated LELVs to promote wound healing and attenuate inflammatory responses, particularly in an LPS-induced inflammation model. Exosome-like natural nanoparticles derived from plants have recently gained attention for their involvement in key biological processes, including cell communication and tissue regeneration. Our results provide evidence supporting the beneficial effects of plant-derived exosomes, particularly LELVs, in mitigating inflammation and enhancing tissue repair.

LELVs were shown to significantly enhance fibroblast proliferation and migration, key processes required for effective wound healing. The increase in cellular proliferation observed at the wound edges following LELV treatment underscores their potential for activating regenerative processes. This suggests that LELVs could serve as an effective therapeutic tool for enhancing wound healing, especially in cases involving dysregulated inflammation, such as LPS-induced wounds. Inflammatory responses are essential components of wound healing; however, excessive or prolonged inflammation can impair healing by disrupting neovascularization and macrophage function [21, 22]. Our findings demonstrated that LELVs reduce LPS-induced inflammation, as evidenced by a marked reduction in TNF-α levels, a key pro-inflammatory cytokine [23, 24]. By modulating the inflammatory response, LELVs foster a more favorable environment for wound healing, facilitating both wound closure and tissue regeneration.

The application of LELVs in LPS-treated wounds mitigated delayed healing and significantly enhanced cell proliferation at the wound edges. These observations suggest that specific bioactive molecules within LELVs may play a role in modulating cellular responses and promoting tissue regeneration. Although this study focused on functional outcomes, future work will involve comprehensive molecular analysis, such as proteomics, RNA sequencing and metabolomics, to identify key components in LELVs. Identifying these bioactive molecules will provide insights into the molecular pathways responsible for the anti-inflammatory and regenerative effects of LELVs.

Our results align with previous studies on plant-derived exosomes [25, 26], which reported similar effects on accelerating wound closure and improving healing outcomes in impaired wound models [27, 28]. The therapeutic potential of plant-derived vesicles like LELVs, combined with their natural origin and ease of production, strengthens the growing body of evidence supporting their biomedical applications. The ability of LELVs to regulate inflammation and stimulate fibroblast activity underscores their promise for wound management.

LELVs offer several advantages over synthetic nanomaterials and mammalian exosomes. Plant-derived exosomes, such as those from *Lithospermum erythrorhizon*, are not only abundant and easy to produce, but also exhibit rapid biodegradability and low immunogenicity [29, 30]. These traits make them particularly effective in crossing cellular membranes and delivering therapeutic agents directly to target cells [31-33]. As a result, LELVs represent a promising alternative for treating a range of inflammatory conditions and promoting wound healing.

Despite these promising results, several challenges remain in advancing LELV therapy toward clinical application. One primary limitation is the relatively low yield of vesicle production from plant sources, which may hinder scalability [34, 35]. Additionally, the absence of classical exosomal markers in plant-derived exosomes complicates their characterization, emphasizing the need for more sophisticated methods to identify and validate these vesicles [36]. Optimizing production processes to improve yield and consistency will be crucial for future applications. Further research is also needed to explore whether similar bioactive components exist in vesicles derived from other plant species, which may broaden the scope of their therapeutic use.

In summary, this study highlights the potential of LELVs as a novel therapeutic strategy for modulating inflammation and enhancing wound healing. The ability of LELVs to reduce LPS-induced inflammation, promote fibroblast proliferation, and accelerate wound closure in an LPS-induced wound healing model suggests that LELVs can complement existing wound healing therapies. Further research into their scalability and characterization will be essential for advancing LELVs into clinical applications in wound care and other inflammatory diseases. With a deeper understanding of their molecular composition and mechanisms of action, LELVs may provide an innovative plant-based solution for treating complex inflammatory conditions.
